# Comparative Analysis of Lycopene Content from Different Tomato-Based Food Products on the Cellular Activity of Prostate Cancer Cell Lines

**DOI:** 10.3390/foods8060201

**Published:** 2019-06-10

**Authors:** Nathalia da Costa Pereira Soares, Monique de Barros Elias, Clara Lima Machado, Bruno Boquimpani Trindade, Radovan Borojevic, Anderson Junger Teodoro

**Affiliations:** 1 Food Science Department, Chemistry Institute, Universidade Federal do Rio de Janeiro (UFRJ), Avenida Athos da Silveira Ramos 149—Cidade Universitária, Rio de Janeiro 21941-909, Brazil; ncpsoares@gmail.com; 2 Nutritional Biochemistry Core, Laboratory of Functional Foods, Universidade Federal do Estado do Rio de Janeiro (UNIRIO), Avenida Pasteur 296—Urca, Rio de Janeiro 22290-240, Brazil; moniquebarros.nutri@gmail.com (M.d.B.E.); claramachado55@gmail.com (C.L.M.); bboquimpani@gmail.com (B.B.T.); 3 Regenerative Medicine Centre, Faculdade de Medicina de Petrópólis (FASE), Avenida Barão do Rio Branco 1003—Petrópolis, Rio de Janeiro 25680-120, Brazil; rrborojevic@gmail.com

**Keywords:** tomato-based food products, lycopene, prostate cancer, bioactive compounds, chemoprevention

## Abstract

Lycopene is more bioavailable in processed tomato products than in raw tomatoes, since arrangement of cis-isomers of lycopene during food processing and storage may increase its biological activity. The aim of the study is evaluate the influence of lycopene content from different tomato-based food products (extract, paste, ketchup and sauce) on cell proliferation, cell cycle, and rate of apoptosis of human prostate cancer cell lines. DU-145 and PC-3 cell lines were treated with lycopene content from different tomato-based food products (500–5000 μg/mL) for 96 h. The data showed a decrease in cell viability in both DU-145 and PC-3 cells after treatment with all lycopene extracts from tomato-based food products. Analysis of cell cycle revealed a decrease in the percentage of prostate cancer cells in G_0_/G_1_ and G_2_/M phases after 96 h of treatment when using lycopene content from tomato paste and tomato extract. However, lycopene extracted from tomato sauce and ketchup promoted a decrease in the percentage of cells in G_0_/G_1_ phase and an increase in S and G_2_/M phases after 96 h of treatment. Lycopene content from all of those tomato-based food products also increased apoptosis in both prostate cancer cell lines. In this regard, lycopene has proved to be a potent inhibitor of cell viability, arrest cell cycle and increase the apoptosis in human prostate cancer cells, suggesting an effect in the balance of human prostate cancer cell lines growth.

## 1. Introduction

Prostate malignant growth (PCa) is the most common cancer and the fifth driving reason for death in men, representing 15% of the absolute analyzed tumors in men and 307,000 deaths, speaking to 6.6% of the all-out male disease mortality. [1]. Many factors included diet, lifestyle, environmental, and genetic factors can contribute to enhance the risk factors for PCa [2]. Several studies found inverse relationships between total fruit and vegetable intake [3] or cruciferous vegetable intake and PCa risk [4,5,6,7,8].

Lycopene is commonly found in tomato products. Due to its lipophilic character, the interactions between lycopene and fats enhance its bioavailability [9]. Therefore, cooking processes using oils in the preparation of tomato sauces and paste are very important [10,11]. In most food sources, lycopene exists predominantly in the all-trans conformation. In contrast, the cis-isomer is thought to provide better bioavailability and might be more easily absorbed [12]. However, lycopene solubilization in warm water collapses of the cell walls; this weakens the connection between the lycopene and the tissue matrix. As a result, lycopene is more accessible and isomerization from the all-trans to the cis conformation is increased [13]. Previous studies have suggested that the bioavailability of lycopene is more substantial in tomato paste than fresh tomatoes. Furthermore, lycopene was found in higher quantities in heated, processed tomato juice than in juice that was unprocessed [14,15]. Thus, in light of the epidemiologically defined cancer-preventing properties of carotenoids, the factors affecting their bioavailability should be taken into account [16].

A number of studies described the association between tomatoes and tomato-based food products with PCa, but the conclusions were unclear [2,17]. The antioxidant activity of lycopene, which is abundant in tomatoes, has also been studied specifically for its influence on PCa [12]. Prospective human studies found that higher lycopene consumption or higher lycopene serum levels were associated with lower PCa risk [18,19]. Two short-term preprostatectomy trials using lycopene supplementation or tomato sauce consumption described the lycopene effect in prostate tissue associated with antioxidant and potentially anticancer properties [20,21]. While several clinical trials suggested an inverse relationship between cancer occurrence and lycopene supplementation [22,23], no large trial study has tested the role of lycopene or tomato-based food products on PCa prevention or treatment [24]. In a cohort of 14,000 Seventh-day Adventist men [25], consumption of only tomatoes, beans, lentils, and peas was found to be statistically significantly related to lower prostate cancer risk in a multivariate analysis. β-carotene food sources seems to be unrelated to risk of prostate cancer. In a larger and more comprehensive dietary study, intake of the carotenoids β-carotene, β-carotene, lutein, and β-cryptoxanthin were not associated with the risk of prostate cancer, but high lycopene intake was related to reduction (26%) in risk [26]. High intake of tomatoes and tomato-based food products, which accounted for highest content of lycopene (82%), reduced risk of prostate cancer by 35% and aggressive prostate cancer by 53% in another study [27].

In vitro, lycopene arrests the cell cycle in several PCa cell lines [28,29,30]. Lycopene-induced delay in progression through the G1 and S phases has also been observed in human cancer cell lines derived from the prostate [31]. Apo-10′-lycopenoic acid and apo-12’-lycopenal, metabolites of lycopene metabolism, [24] can cause cell cycle arrest in cancer cells [32,33]. Cancer cells arrested by serum deprivation in the presence of lycopene cannot return to the cell cycle after serum re-addition [19]. Lycopene and tomato comsumption may modify testosterone metabolism and serum concentrations, and may impact gene expression in human prostate cancer cells, normal rat prostate, and prostate cancer xenografts [19,32,33,34]. In addition to the antioxidant action, lycopene has several biological functions important for human health. Previous studies describes mechanisms in preventing carcinogenesis, including the antiproliferative insulin-like growth factor-1 inhibition, induction of cell differentiation, and apoptosis, connexin, and enhance of gap junctional intercellular communications [35]. For maintaining normal prostate growth and cell renewal, the complex equilibrium between cell growth or differentiation factors and apoptosis inducing factors is critical. Similar to other tissues, prostate is normally composed of cells that divide and replicate in an orderly and controlled manner, however it can likewise contain cells with modified division patterns that give rise to benign or malignant tumors [36].

The treatment of LNCaP cells with physiologically attainable concentrations of lycopene (0.3–3.0 μM) can significantly reduce the mitochondrial transmembrane potential, induce the release of mitochondrial cytochrome c, and increase annexin V binding, compatible with the induction of apoptosis [37]. Soares et al. [30] showed that lycopene induced apoptosis in prostate cancer cells with an average 1.35-fold increase after 48 h treatment, reaching a maximum 2.25-fold increase after 96 h, at the highest lycopene concentration (10 μM). It has been demonstrated that lycopene alters the equilibrium of Bcl-2/Bax expression in PCa cells treated with lycopene. Induction of apoptosis is an important strategy in the tumor suppressive function of p53 [38]. This prompt might be significant for the understanding the effects of lycopene on prostate cancer.

Although the role of lycopene in the prevention of PCa has been studied extensively, human studies examining the role of lycopene in cancer are now being conducted [39]. The majority of studies on the effects of lycopene on cell cycle were performed 48 h after lycopene treatment; this can lead to the underestimation of the effect of this substance.

In the present study, we evaluated the influence of lycopene content from different tomato-based food products (extract, paste, sauce and ketchup) on cell proliferation, cell cycle, and rate of apoptosis of human prostate cancer cell lines (DU-145 and PC-3).

## 2. Materials and Methods

### 2.1. Chemicals, Reagents and Materials

Dulbecco’s cell culture medium (DMEM) and bovine serum albumin were obtained from Sigma, and fetal bovine serum (FBS) from Laborclin (São Paulo, Brazil). Cell culture flasks and plates were procured from Nunc (Roskilde, Denmark). All chemicals were of analytical grade.

### 2.2. Samples and Lycopene Extraction

Tomato-based food products (tomato extract, tomato paste, tomato sauce and ketchup) from Brazilian commercial products were purchased in the local supermarket (Rio de Janeiro, Brazil). Lycopene extracts from each tomato-based product were obtained by conventional solvent extraction using organic solvent (absolute ethanol). A 500 g sample was weighed into a 3-L glass tube with a glass filter bottom (50 mm × 1500 mm), and lycopene was extracted for 40 min with 1 L of absolute ethanol in an ultrasonic bath. Subsequently, the solvent was removed using a vacuum evaporator (Marconi^®^) at 60 °C for 6 h and the material was weighed. After those steps, the lycopene extracts obtained from each tomato-based product were frozen at −78 °C for 24 h. Using a freeze drier (Terroni LD 3000) those extracts described above were submitted to freeze-drying, 20 h, at less than 200 μmHg. The material obtained from this process had a “flour” texture, and it was stored in amber bottles, at −18 °C, until the test procedures with the cell lines [40,41]. The extraction, separation and identification of lycopene were performed using a HPLC (Figure S2).

### 2.3. High Performance Liquid Chromatography (HPLC) Analysis

Profiles of the carotenoids were determined by HPLC (Waters 2695—Alliance Model, Milford, MA, USA) controlled by the Empower software program with the column oven at 33 °C and photodiode array detector (DAD 996-Waters^®^). Carotenoid separation was obtained in a C30 column (S-3 Carotenoid, 4.6 mm × 250 mm, YCMTM) purchased from Waters. The mobile phase HPLC grade solvents were purchased from Tedia (Rio de Janeiro, RJ, Brazil) and consisted of 8:2 (methanol:t-butyl methyl ether, v:v). The flow rate was 0.8 mL/min and the injection volume samples was 15 µL. Analysis running time was 28 min. All analyses were performed in triplicate. Carotenoids were identified based on their retention times and ultraviolet–visible (UV/Vis) absorption spectra, compared to the retention times of the carotenoid standards. The carotenoid standards were obtained from Sigma-Aldrich. The identification of all-trans lycopene and cis isomers was done by elution order and UV–vis spectra; they were compared to the published data [40,41] (Figure S1). All the solvents and chemicals were purchased from Sigma and Merck. Lycopene standard (90% all-E-lycopene) were obtained from Sigma Aldrich (USA).

### 2.4. Cell Culture Experiments

Cell lines were obtained from the Rio de Janeiro Cell Bank (BCRJ), which certified their identity and quality (Inmetro, Rio de Janeiro, RJ, Brazil). The BCRJ ensures that all cell cultures undergo microbiological analyzes and tests for mycoplasma detection before being sent to the customers. Prostate cancer cell lines DU-145 (BCRJ code: 0078) and PC-3 (BCRJ code: 0269) were grown in 25 cm^2^ cell culture flasks at a density of 4.0 × 10^6^ cells/flask and in a 37 °C humidified incubator with 5% carbon dioxide (CO2) (Thermo Scientific CO_2_ Incubator). Cells were grown in DMEM supplemented with 10% fetal bovine serum (FBS) and 2 g/L HEPES (4-(2-hydroxyethyl)-1-piperazineethanesulfonic acid) buffer, pH 7.4. Cells were handled in safe conditions that meet sterilization procedures standards, using a biosafety cabinet to its manipulation. The cabinet was irradiated for 30 min prior to use and all the surfaces were sterilized using EtOH 70%. Cells were passaged by trypsinization when they reached 70–80% confluence, about twice a week. The experiments have been started after the sixth passage. For each experiment, all cells were plated at a density of 10^4^ cells/cm^2^ in 6 and 96-multiwell plates for cell cycle and cell proliferation analyses, respectively. Lycopene extracts were dissolved in water at 50 °C within a range from 500 to 5000 μg/mL and depending on the lycopene concentration of each product, the extract was normalized to in all extracts the concentration of lycopene in the stock solution having the same concentration of lycopene between the different extracts. Lycopene extracts from the different tomato products were then added to the plates. Different plates were used for each lycopene extract and experimental cells were included on the same plate as control cells. Cells were then incubated for 96 h with daily medium replacement.

### 2.5. Cell Viability Assay

The anticancer activity of extracts on DU-145 and PC-3 cells were determined by the MTT (3-(4, 5-dimethyl thiazol-2yl)-2, 5-diphenyl tetrazolium bromide) assay was used to assess the cytotoxicity Cells (1 × 10^4^/well) were plated in 0.2 ml of medium/well in 96-well plates for 24 h. For MTT assay the medium from the wells was removed carefully after incubation. After treatment with lycopene extracts from the different tomato-based food products and incubation for 96 h (six wells for each sample), 20 μL of MTT (5 g/L) were added to each well. After incubation, the medium was removed and 100 μL/well sodium dodecyl sulfate (SDS). Presence of viable cells was visualized by the development of purple color due to formation of formazan crystals. The suspension was transferred to enzyme-linked immunosorbent microplate reader (Bio-Rad 2550) at 490 nm. Measurements were performed and cellular viability inhibition rate (CVIR) was calculated using the following formula: CVIR= (1 — average absorbance value of experimental group/average absorbance value of control group) × 100%.

### 2.6. Cell Cycle Analysis

Briefly, cells were washed with calcium and magnesium-free phosphate-buffered saline (PBS) and detached from cell culture flasks with trypsin at 37 °C. The cells were washed twice with PBS, and 1 × 10^6^ cells were resuspended in 1.0 mL of ice-cold VindeLov solution containing 0.1% Triton X-100, 0.1% citrate buffer, 0.1 mg/mL RNase, and 50 μg/mL propidium iodide (Sigma Chemical Co., St. Louis, MO, USA). Flow cytometry was used to determine the effect of the extracts on the cell cycle using a FACSCalibur flow cytometer (Becton Dickinson, Mountain View, CA, USA). The relative proportions of cells with diploid DNA content were acquired and analyzed using CellQuest and WinMDI 2.9 software, respectively. Results are expressed as percentage of cells in each phase of cell cycle determined with EXPO32 V1.2 analysis software (Beckman Coulter, Inc., Brea, CA, USA). The cell dissociation procedure did not affect fluorescence under the experimental conditions that were used in this study.

### 2.7. Apoptosis Assay

DU-145 and PC-3 prostate cancer cell lines were treated with lycopene extracts, at concentrations of 500 and 5000 μg/mL, in 6-well plates. After 96 h of incubation, the non-adherent cells were collected, and adherent cells were quickly washed with PBS and detached with trypsin/EDTA(ethylenediamine tetraacetic acid) 0.125% (Sigma Chemical Co., Saint Louis, MO, USA) at room temperature. Cells were centrifuged to remove the medium, washed with PBS and stained with Annexin V-FITC and PI in binding buffer (BD Pharmingen) according to the manufacturer’s instructions. Stained cells were analyzed using a FACSCalibur (BD Bioscience, Franklin Lakes, NJ, USA) and analyzed using WinMDI 2.9 software. Data were reported as the percentage of apoptosis, obtained by determining the numbers of apoptotic cells versus the total numbers of cells.

### 2.8. Statistical Analysis

Results were expressed as mean ± SD for a given number of three independent experiments done in duplicate. Data were analyzed by using one way analysis of variance (ANOVA) followed by the Tukey test using the Graph Pad Prism 5.0 and Statistical 6.0 program (company, city, country (version 5.04, GraphPad Software, San Diego, CA, USA). Statistical differences were considered significant when the value was *p* < 0.05.

## 3. Results and Discussion

### 3.1. HPLC Analysis

Table 1 summarizes the data concerning, total carotenoid content, cis-lycopene and all-trans-lycopene isomers from the analyzed tomato-based food products. The mean lycopene content was 96.65% in tomato sauce, 96.48% in ketchup, 95.12% in tomato extract, and 97.78% in tomato paste. However, the lycopene content of tomato sauce was not statistically different from that of ketchup and tomato extract. Among the samples analyzed, ketchup displayed a higher content of cis-lycopene (9.20 μg/g). These values are similar to those reported by Barber and Barber [42], as well as Waliszewski and Blasco [43].

Cis-lycopene-rich tomato sauce has higher bioavailability than trans-lycopene-rich tomato sauce in healthy adult subjects. Cis-isomers of lycopene are produced during processing and cooking of tomato products [44,45]. It is conceivable that all-trans-lycopene, a long linear molecule, may be less soluble in bile acid micelles. Lycopene in fresh tomatoes occurs mostly in the trans-form. In contrast, cis-isomers of lycopene may move more efficiently across plasma membranes and preferentially incorporate into chylomicrons [46]. However, is still unclear data for metabolism, biotransformation, distribution, and biological relevance of the cis-isomers of carotenoids in human tissues. Differential absorption, transport, and uptake of specific stereoisomers can also be hypothesized [47,48]. Further endeavors to explain the structures of geometric lycopene isomers and biological mechanisms in the prostate may prompt the advancement of novel chemopreventive agents.

### 3.2. Effect of Lycopene Extracts on the Number of Viable Cells in Culture

Our study provides evidence that lycopene in tomato products may inhibit the growth of human PCa cells. The human prostate contains lycopene and other dietary carotenoids, supporting the hypothesis that tomato-derived carotenoids may directly impact the prostate. Prostate cancer cell lines (PC-3 and DU-15) were derived from distant metastases of. Accordingly, they have gone through the epithelial-mesenchymal progress and are expected to be different, both from primary prostate cancer and from each other, since each established cancer line passes through extensive selection both in vivo and in subsequent culture in vitro. DU-145 cells displayed a higher inhibition of proliferation in elevated levels of lycopene compared to the PC-3 cell line.

**Figure 1:**
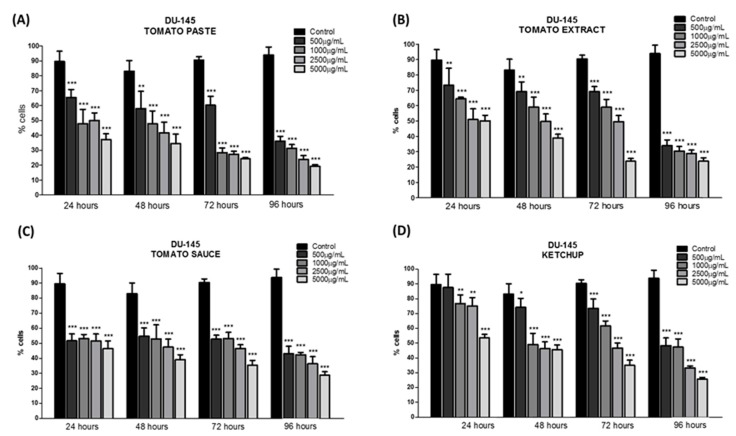
Effect of lycopene obtained of tomato paste (**A**), tomato extract (**B**), tomato sauce (**C**), and ketchup (**D**) on DU-145 cell viability after 24, 48, 72, and 96 h of exposure, respectively. The results are expressed as mean ± error standard and significant differences between untreated cells (Control) and those treated with lycopene (500, 1000, 2500, and 5000 µg/mL) were compared by one-way ANOVA followed by Tukey’s multiple comparison post-hoc test. * *p* < 0.05; ** *p* < 0.01; *** *p* < 0.001.

**Figure 2:**
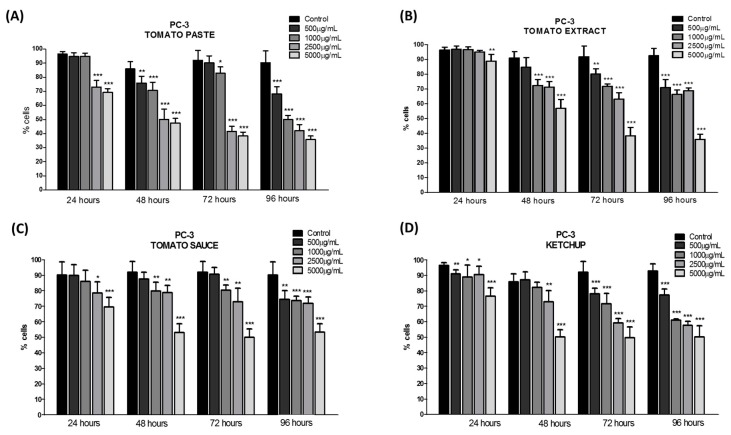
Effect of lycopene obtained of the tomato paste (**A**), tomato extract (**B**), tomato sauce (**C**), and ketchup (**D**) on DU-145 cell viability after 24, 48, 72, and 96 h of exposure, respectively. The results are expressed as mean ± error standard and significant differences between untreated cells (Control) and those treated with lycopene (500, 1000, 2500, and 5000 µg/mL) were compared by one-way ANOVA followed by Tukey’s multiple comparison post-hoc test. * *p* < 0.05; ** *p* < 0.01; *** *p* < 0.001.

Both cell lines showed the normal growth characteristics expected under standard in vitro conditions. The plating of cancer cell lines was followed by a 24 h recovery period, and cells were subsequently incubated with 500, 1000, 2500, and 5000 μg/mL of lycopene extracts for 24, 48, 72, and 96 h. Using the MTT assay, we observed a decrease in cell viability in both the cancer cell lines after treatment with all extracts of tomato-based food products. Even after only 24 h of treatment, lycopene promoted an average inhibition of 35% for DU-145 cells, which increased to 55% after 96 h of treatment for all tomato-based food products (Figure 1). Lycopene treatment inhibited viability of PC-3 cells by approximately 40%, after 96 h (Figure 2). A potent inhibitory effect on PC-3 cell viability was observed using lycopene extracted from tomato paste, and the highest reductions in cell viability were achieved even with 24 h of incubation. No statistically significant differences were observed between the effect of lycopene content from tomato-based food products after 96 h of treatment in both the cell lines.

DU-145 cells, which are moderately aggressive, exhibited a greater inhibition of proliferation at high levels of lycopene compared to PC-3 cells, which are highly aggressive. The lycopene effect was found to be time-dependent, because this effect required a relatively long incubation time to achieve improved action.

### 3.3. Effect of Lycopene Extracts on Cell-Cycle Progression

**Figure 3:**
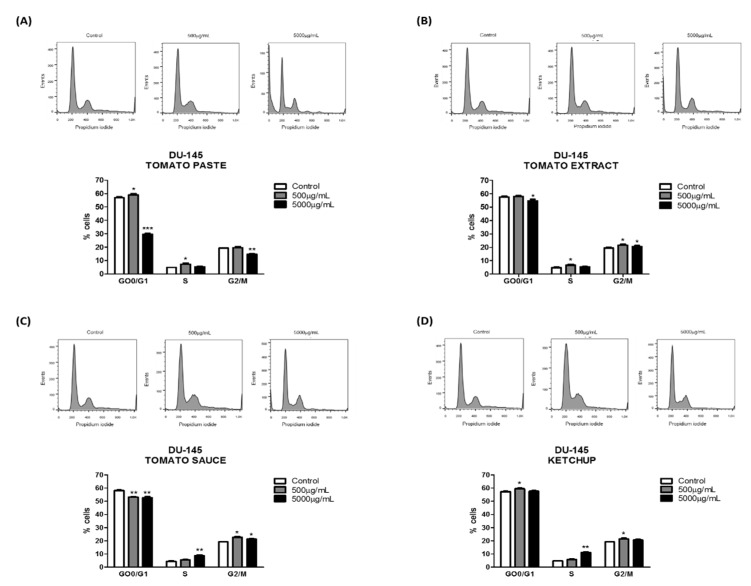
Effect of lycopene obtained from tomato paste (**A**), tomato extract (**B**), tomato sauce (**C**), and ketchup (**D**) on DU-145 cell cycle after 96 h of exposure, respectively. The results are expressed as mean ± error standard and significant differences between untreated cells (Control) and those treated with lycopene (500 and 5000 µg/mL) were compared by 1-way ANOVA followed by Tukey’s multiple comparison post-hoc test. * *p* < 0.05; ** *p* < 0.01; *** *p* < 0.001.

**Figure 4:**
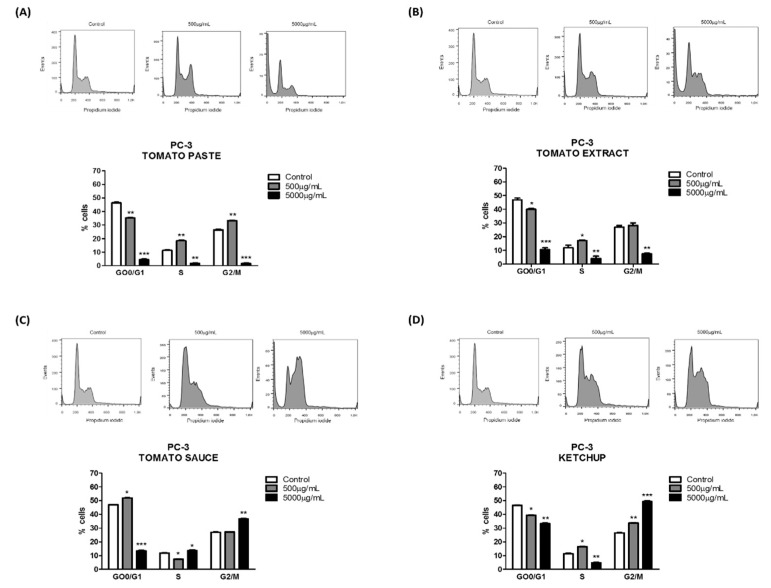
Effect of lycopene obtained of tomato paste (**A**), tomato extract (**B**), tomato sauce (**C**), and ketchup (**D**) on PC-3 cell cycle after 96 h of exposure. The results are expressed as mean ± error standard and significant differences between untreated cells (Control) and those treated with lycopene (500 and 5000 µg/mL) were compared by 1-way ANOVA followed by Tukey’s multiple comparison post-hoc test. * *p* < 0.05; ** *p* < 0.01; *** *p* < 0.001.

We treated cells with lycopene for 96 h and quantified the percentage of cells in the different cell-cycle phases to elucidate the mechanism by which lycopene regulated cell growth. Flow cytometry analysis of cell cycle revealed that lycopene extracted from tomato paste (5000 µg/mL) decreased the percentage of DU-145 cells in G_0_/G_1_ and G_2_/M phases (Figure 3A). However, lycopene extracted from tomato sauce and tomato extract (Figure 3B,C) decreased the percentage of cells in G_0_/G_1_ phase and increased in S and G_2_/M phases (5000 µg/mL) after 96 h of treatment. In addition, lycopene extracted from ketchup (500 µg/mL) increased the percentage of cells in G_0_/G_1_ and G_2_/M phases (Figure 3D). No changes were observed when using 5000 µg/mL lycopene extracted from ketchup. In PC-3 cells, a significant reduction in the percentage of cells in the G_0_/G_1_ and G_2_/M phases was achieved with lycopene extracted from tomato paste and tomato extract, when used at higher concentration (Figure 4A,B). The effect of lycopene extracted from tomato sauce and ketchup promoted an accumulation of PC-3 cells in the G_2_/M phase (Figure 4C,D).

In both cell lines, the cell cycle analysis showed that lycopene reduced the percentage of cells in G_0_/G_1_ and G_2_/M phases after 96 h of treatment in the metastatic PCa cell lines, using lycopene extracted from tomato paste and tomato extract. Although the arrest of cells in G_0_/G_1_ phase can be reverted, and cells can proceed with proliferation after interruption of the treatment, G_2_/M arrest potentially leads to apoptosis. However, lycopene extracted from tomato sauce and ketchup decreased the percentage of cells in G_0_/G_1_ phase and increased the percentage of cells in S and G_2_/M phases after 96 h of treatment. The cell cycle arrest in G_2_/M phase is vital, because it leads to apoptosis when cells cannot recover and proceed to cell division.

Furthermore, considering that lycopene extracted from all tomato-based food products interfered with cell cycle and cell viability, it was important to investigate whether these products disturbed apoptosis during the in vitro treatment. DU-145 and PC-3 cells displayed a significant increase in apoptosis, suggesting that another mechanism may be involved. According to Renju et al. [49], exposure of PC-3 and DU-145 cell lines to lycopene isolated from Chlorella marina at a dose of 20 and 50 μM significantly inhibited cell growth, and apoptosis was strongly induced at 50 μM, demonstrating the anti-proliferative and apoptotic effects of lycopene. Cell-cycle deregulation is an important step in cancer development [49]. Previous studies reported that lycopene induced cell cycle arrest in G_1_/S phases, which was mediated by the downregulation of cyclins E and D1, and/or by the upregulation of cyclin A and p27 [50].

### 3.4. Apoptosis

It has already been elucidated that apoptosis may modulate the malignant phenotype, and studies have uncovered that a high recurrence of apoptosis was seen in unexpectedly relapsing tumors and in tumors treated with cytotoxic anticancer agents [51]. Hence, many studies have been led to demonstrate the impact of tomato-based products on inducing programmed cell death [52,53].

**Figure 5:**
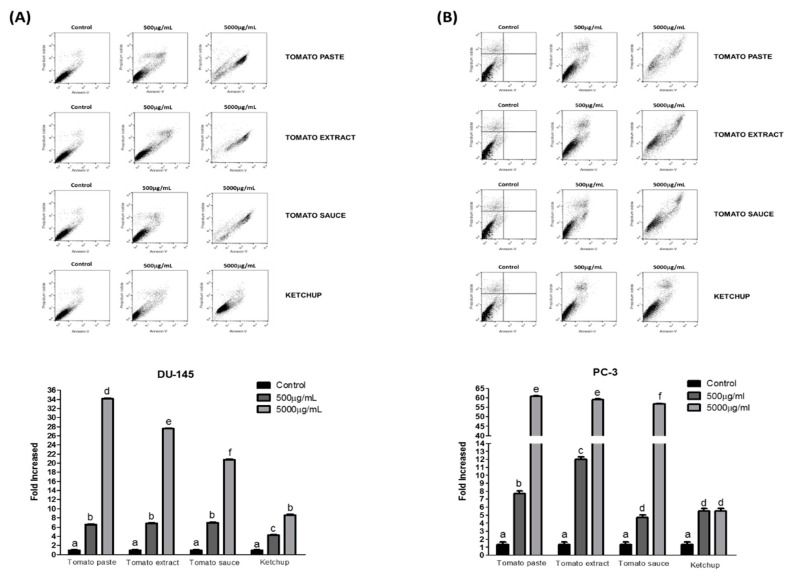
The effect of lycopene obtained from tomato paste, tomato extract, tomato sauce and ketchup in the process of programmed death in DU-145 (**A**) and PC-3 (**B**) cells the after 96 h treatment. The flow cytometric analyses are shown according to the exposure time and carotenoid concentration. The quantitative results of lycopene on cell lines are shown after 96 h. The data are expressed as mean ± standard error. Variation in the letters between samples indicates significance difference (*p* < 0.05).

Apoptosis induction was reported by annexin V and PI biomarkers. Altering the balance between proliferation and apoptosis is associated with cancer, and quantification of apoptosis can be a useful measure of cancer cell kinetics. We evaluated the effect of lycopene from all tomato-based products after 96 h of incubation on different stages of the cell death process of DU-145 and PC-3 cells. Table 2 shows the percentage of viable, early apoptotic, late apoptotic, and necrotic cells treated with lycopene extracts from tomato paste, tomato extract, tomato sauce, and ketchup (500 and 5000 μg/mL). Figure 5 shows the influence of lycopene extracts from tomato-based products on the rate of apoptosis.

In DU-145 cells, after 96 h of incubation, tomato paste extract showed an increase upto 34.1 times (5000 µg/mL) in the population of apoptotic cells (early and late apoptosis) compared to control (Figure 5A). Ketchup extract promoted a lower effect from all the tomato-based product extracts analyzed, achieving an increase up to 8.6 times in apoptotic cells, with the highest lycopene concentration (5000 µg/mL). In PC-3 cells (Figure 5B), no statistical difference was observed between lycopene extracts from tomato paste, tomato extract, and tomato sauce at higher treatment concentrations. A potent effect was achieved with a relative increase rate of 58.9 times in the population of apoptotic cells (early and late apoptosis). However, for this cell line, ketchup extract also produced a lower effect compared to all the tomato-based products analyzed, achieving an increase of up to 5.5 times in apoptotic cells.

Increase in early and late apoptotic cells was observed in DU-145 and PC-3 cells treated with extracts from all tomato-based products and both concentrations of lycopene, but the most notable effect was produced by tomato paste extract (Table 2). Despite the unknown comparative bioavailability values for lycopene from various tomato products, lycopene from processed tomato products appears to be more bioavailable than raw tomatoes. The release of lycopene from the food matrix due to processing, the presence of dietary lipids and heat-induced isomerization from all-trans to cis enhance the bioavailability of lycopene [54].

Recognizing that current evidence of dietary intake and blood concentrations of lycopene reflects consumption of tomatoes and tomato products rather than purified supplements of lycopene is critical. The pharmacokinetic properties of lycopene remain poorly understood. Further research on this potentially important carotenoid’s bioavailability, pharmacology, and biology is clearly warranted. Until more definitive information is available on the specific benefits of purified forms of lycopene, current recommendations should highlight the health benefits of diets rich in a variety of fruits and vegetables, including tomatoes and tomato-based products [55,56].

## 4. Conclusions

Our results may contribute to a better understanding of the potential role of tomato-based products, which are a readily available source of lycopene. The products displayed potent anti-carcinogenic effects against PC-3 and DU-145 cells. Lycopene obtained from tomato paste and extract decreased the percentage of PCa cells in G_0_/G_1_ and G_2_/M phases after 96 h of treatment. However, tomato sauce and ketchup extract decreased the percentage of cells in G_0_/G_1_ phase and increased percentage of cells in S and G_2_/M phases after 96 h of treatment. Lycopene also increased apoptosis in both PCa cell lines. These data show that tomato lycopene inhibits cell proliferation, arrests cell cycle in different phases, and increases apoptosis in human PCa cell lines. Thus, tomato lycopene may constitute the basis of new therapeutic strategies for the treatment of prostate malignancy.

In conclusion, the present study supports the proposal that all-trans-lycopene and lycopene extracted from tomato-based food products may have a protective effect on PCa. These findings add further support to current dietary recommendations to increase consumption of food sources of lycopene to reduce prostate cancer risk.

## Figures and Tables

**Table 1:** Total carotenoid content, cis-lycopene, and all-trans-lycopene isomers from the analyzed tomato-based food products.

Sample	Total Carotenoids	*Cis*-Lycopene	*Trans*-Lycopene	Lycopene Content
	(μg/g)	(μg/g)	(μg/g)	(%)
**Ketchup**	147.81 ± 8.35 ^a^	9.20 ± 0.89 ^a^	133.39 ± 6.64 ^a^	96.48 ± 0.36 ^a^
**Tomato Extract**	85.60 ± 1.09 ^b^	6.48 ± 0.88 ^b^	74.94 ± 1.73 ^b^	95.12 ± 0.22 ^b^
**Tomato Sauce**	168.95 ± 5.36 ^c^	7.40 ± 0.49 ^b^	155.94 ± 7.45 ^c^	96.65 ± 1.63 ^a,b,c^
**Tomato Paste**	77.57 ± 1.81 ^d^	5.05 ± 0.40 ^b^	70.80 ± 2.09 ^b^	97.78 ± 0.10 ^c^

Lycopene content (%) was measured through the following formula: (cis-lycopene + trans-lycopene) × 100/total carotenoids. The data are expressed as mean ± SD. Variation in the letters between samples indicates significant difference (*p* < 0.05).

**Table 2:** Effect of tomato-based products on different cell death stages in human prostate cancer cell lines (DU-145 and PC-3) after 96 h.

Compounds										
Cell Type	Cell Death Stages	Untreated Cells (Control)	Tomato Paste		Tomato Extract		Tomato Sauce		Ketchup	
			500 μg/mL	5000 μg/mL	500 μg/mL	5000 μg/mL	500 μg/mL	5000 μg/mL	500 μg/mL	5000 μg/mL
**DU-145**	Viable cells	93.33 ± 1.23 ^a^	89.10 ± 2.71 ^b^	64.30 ± 1.56 ^c^	89.36 ± 1.48 ^b^	70.93 ± 2.02 ^d^	88.45 ± 1.48 ^b^	78.45 ± 1.20 ^e^	91.32 ± 1.24 ^a^	89.80 ± 0.32 ^b^
	Early apoptosis	0.49 ± 0.12 ^a^	2.32 ± 0.53 ^b^	13.20 ± 1.70 ^c^	2.12 ± 0.29 ^b^	8.71 ± 0.40 ^d^	3.56 ± 0.56 ^b^	6.65 ± 0.35 ^e^	1.14 ± 0.28 ^f^	2.90 ± 0.30 ^b^
	Late apoptosis	0.57 ± 0.14 ^a^	4.35 ± 1.09 ^b^	21.05 ± 0.64 ^c^	4.82 ± 0.54 ^b^	19.00 ± 0.85 ^c^	3.50 ± 0.34 ^b^	14.25 ± 0.64 ^d^	3.22 ± 0.46 ^b^	5.90 ± 0.25 ^e^
	Necrosis	5.61 ± 0.97 ^a^	4.23 ± 1.06 ^b^	1.45 ± 0.49 ^b^	3.70 ± 0.64 ^c^	1.36 ± 0.38 ^b^	4.49 ± 0.59 ^a^	0.65 ± 0.21 ^d^	4.32 ± 0.50 ^a^	1.40 ± 0.27 ^b^
**PC-3**	Viable cells	97.64 ± 0.08 ^a^	90.26 ± 0.23 ^b^	37.37 ± 0.04 ^c^	87.67 ± 0.89 ^b^	40.05 ± 0.49 ^d^	93.39 ± 0.04 ^b^	42.78 ± 0.37 ^c^	93.73 ± 0.37 ^b^	94.14 ± 0.18 ^b^
	Early apoptosis	0.84 ± 0.07 ^a^	1.87 ± 0.15 ^b^	21.53 ± 0.33 ^c^	3.07 ± 0.22 ^d^	16.35 ± 0.35 ^e^	1.87 ± 0.20 ^b^	18.35 ± 0.35 ^e^	1.64 ± 0.28 ^b^	2.86 ± 0.29 ^d^
	Late apoptosis	0.82 ± 0.07 ^a^	5.54 ± 0.35 ^b^	39.00 ± 0.57 ^c^	8.63 ± 0.48 ^d^	42.52 ± 0.35 ^c^	2.55 ± 0.49 ^e^	38.15 ± 0.64 ^c^	3.58 ± 0.49 ^e^	2.36 ± 0.32 ^e^
	Necrosis	0.7 ± 0.06 ^a^	2.33 ± 0.39 ^b^	2.10 ± 0.28 ^b^	0.63 ± 0.19 ^a^	1.08 ± 0.21 ^c^	2.19 ± 0.25 ^b^	0.72 ± 0.21 ^a^	1.05 ± 0.16 ^a^	0.64 ± 0.13 ^a^

Legend: the cell-cycle phases and quantitative results are illustrated in accordance with the exposure time and carotenoid concentration. The experiment is expressed as mean ± standard error. Small different letters indicate significant differences (*p* < 0.05) within same line among different concentrations versus control group.

## Supplementary Materials

The following are available online at https://www.mdpi.com/2304-8158/8/6/201/s1, Figure S1: HPLC analysis of lycopene standard and UV spectrum of all-trans-lycopene (**A**) and cis-lycopene (**B**); Figure S2. HPLC analysis of tomato extract (**A**), tomato paste (**B**), tomato sauce (**C**), and ketchup (**D**).
