# Supplementary material for: Comparative Analysis of Lycopene Content from Different Tomato-Based Food Products on the Cellular Activity of Prostate Cancer Cell Lines
Source: Foods. 2019 Jun 10;8(6):201. doi: 10.3390/foods8060201 (PMC6617171; doi:10.3390/foods8060201)
Supplement: Supplementary file 1 [file foods-08-00201-s001.pdf]

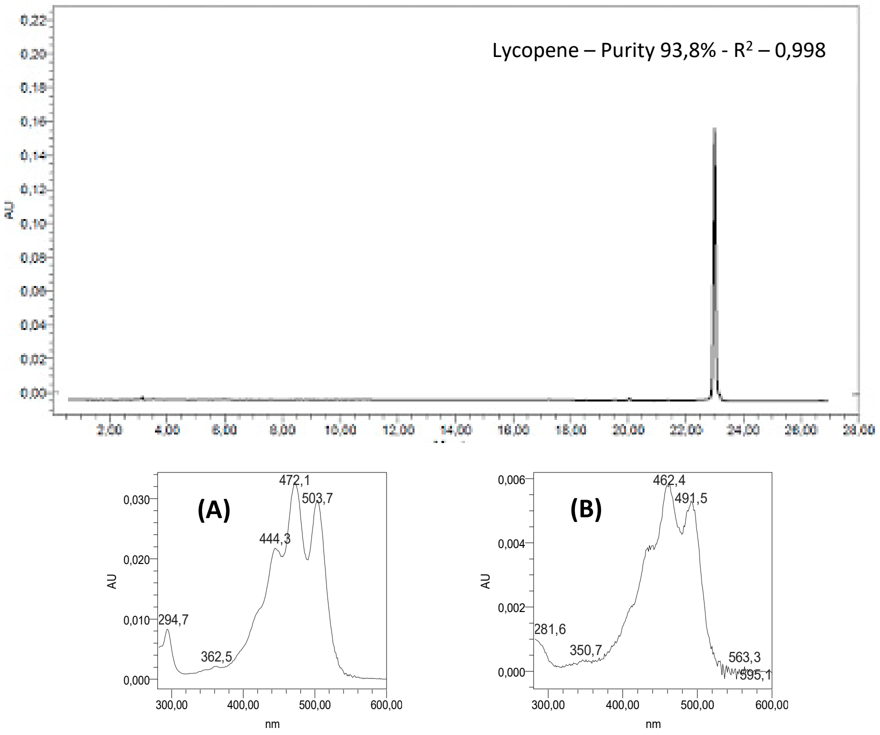

Figure S1. HPLC analysis of lycopene standard and UV spectrum of all-trans-lycopene (A) and cis-lycopene(B) .

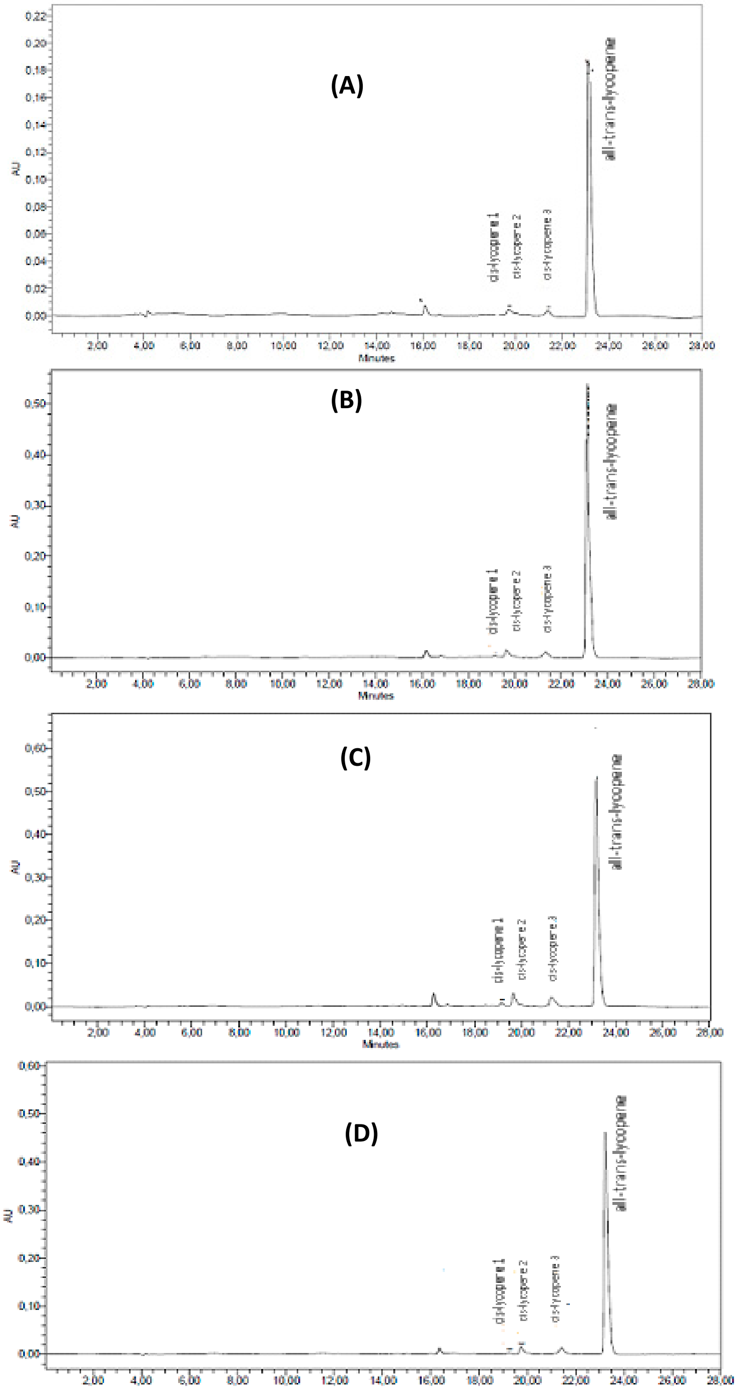

Figure S2. HPLC analysis of Tomato Extract (A), Tomato Paste (B), Tomato Sauce (C) and Ketchup (D).

CERTIFICATE OF ENGLISH EDITING

This document certifies that the paper listed below has been edited to ensure that the language is clear and free of errors. The edit was performed by professional editors at Editage, a division of Cactus Communications.The intent of the author's message was not altered in any way during the editing process. The quality of the edit has been guaranteed, with the assumption that our suggested changes have been accepted and have not been further altered without the knowledge of our editors.

Title Of The Paper

Comparative analysis of lycopene content from different tomato-based food products on the cellular activity of prostate cancer cell lines.

Authors

Nathalia da Costa Pereira Soares, Monique de Barros Elias, Clara Lima Machado, Bruno Boquimpani Trindade, Radovan Borojevic, Anderson Junger Teodoro

Job Code

ANEOD_4

editage by CACTUS

Signature

Vikas Narang

Vikas Narang,
Senior Vice President,
Operations-Author Services, Editage

Date of Issue
April 29, 2019

Editage, a brand of Cactus Communications, offers professional English language editing and publication support services to authors engaged in over 500 areas of research. Through its community of experienced editors, which includes doctors, engineers, published scientists, and researchers with peer review experience, Editage has successfully helped authors get published in internationally reputed journals. Authors who work with Editage are guaranteed excellent language quality and timely delivery.

INFORMATION SECURITY MANAGEMENT SYSTEM DNV·GL ISO/IEC 27001

CACTUS.

Contact Editage

Worldwide	Japan	Korea	China	Brazil	Taiwan
request@editage.com	submissions@editage.com	submit-korea@editage.com	fabiao@editage.cn	contato@editage.com	submitjobs@editage.com
+1 877-334-8243	+81 03-6868-3348	1544-9241	400-005-6055	0800-892-20-97	02 2657 0306
www.editage.com	www.editage.jp	www.editage.co.kr	www.editage.cn	www.editage.com.br	www.editage.com.tw

Figure S3. Certificate of English Editing
